# Reliable and Fast Genotyping Protocol for Galactosylceramidase (Galc) in the Twitcher (Twi) Mouse

**DOI:** 10.3390/biomedicines10123146

**Published:** 2022-12-06

**Authors:** Sara Carpi, Ambra Del Grosso, Miriam De Sarlo, Laura Colagiorgio, Luca Scaccini, Ilaria Tonazzini, Gabriele Parlanti, Marco Cecchini

**Affiliations:** NEST, Istituto Nanoscienze-CNR and Scuola Normale Superiore, Piazza San Silvestro 12, 56127 Pisa, Italy

**Keywords:** SNP genotyping, Krabbe, *Galc*, allelic discrimination, Twitcher, globoid cell leukodystrophy

## Abstract

Twitcher (Twi) is a neurological Krabbe disease (KD, or globoid cell leukodystrophy) spontaneous mutant line in mice. The genome of the Twi mouse presents a single nucleotide polymorphism (SNP), leading to an enzymatically inactive galactosylceramidase (Galc) protein that causes KD. In this context, mouse Twi genotyping is an essential step in KD research. To date, the genotyping method used is labor-intensive and often has ambiguous results. Here, we evaluated a novel protocol for the genotype determination of *Galc* mutation status in Twi mice based on the allele-discrimination real-time polymerase chain reaction (PCR). Here, DNA is extracted from Twi mice (*n* = 20, pilot study; *n* = 120, verification study) and control group (*n* = 10, pilot study; *n* = 30 verification study) and assessed by allele-discrimination real-time PCR to detect SNP c.355G>A. Using the allele-discrimination PCR, all of the samples are identified correctly with the genotype GG (wild-type, WT), GA (heterozygote, HET), or AA (homozygote, HOM) using the first analysis and no animals are not genotyped. We demonstrated that this novel method can be used to distinguish KD timely, accurately, and without ambiguity in HOM, WT, and HET animals. This protocol represents a great opportunity to increase accuracy and speed in KD research.

## 1. Introduction

Krabbe disease (KD or globoid cell leukodystrophy; Online Mendelian Inheritance in Man (OMIM) no. 245200), is a lysosomal storage disease, a group of inherited metabolic disorders caused by enzyme deficiencies within the lysosome. KD is caused by a deficiency of the acid hydrolase galactosylceramidase (GALC; EC 3.2.1.46). GALC is responsible for the degradation of galactosylceramides and other sphingolipids that are abundant in myelin membranes. The absence of GALC leads to the toxic accumulation of galactosylsphingosine (psychosine, PSY). PSY accumulates primarily in oligodendrocytes and Schwann cells, resulting in progressive and profound demyelination of the central and peripheral nervous systems [1].

Despite enormous effort, there is no cure for KD, and the current standard of care is mostly supportive [2]. However, research on novel treatment strategies is ongoing, and some appear to have promising results. In this context, the existence of a spontaneously arising mouse model of KD, the Twitcher (Twi) [3], represents a great opportunity to better understand the pathogenesis of the disease and to test innovative therapeutic strategies.

The genome of the Twi mouse presents a specific nucleotide substitution, which is defined as a sequence change where, compared to a reference sequence, one nucleotide is replaced by another nucleotide [4]. In detail, the single nucleotide substitution of the G nucleotide at codon 355 for A (c.355G>A) changes it from tryptophan to a stop codon (p.W355*) [5]. The single nucleotide substitution in the *Galc* gene on both alleles creates a premature stop codon, resulting in premature termination of the transcription and leading to an enzymatically inactive Galc fragment [6,7]. Possible genotypes are GG (wild-type, WT), GA (heterozygote, HET), and AA (homozygote, HOM).

Indeed, mouse genotyping is an essential step in animal research because it allows for the identification of animals that will be bred to generate and maintain a colony (HET), or be used in approved experiment study protocols on KD (HOM). The consequences of genotyping errors and animal misidentification should not be underestimated and must be controlled through efficient and robust PCR genotyping [4,8,9].

Here, we tested (in a pilot study) and validated (in verification study) a rapid, accurate, high-throughput, unambiguous allele-discrimination real-time polymerase chain reaction (PCR) protocol for genotype determination of *Galc* mutation status in Twi mice.

## 2. Materials and Methods

### 2.1. Animal Procedures

Twi heterozygous mice (HET_Twi) (TWI+/− C57BL6 mice; the Jackson Laboratory, Sacramento, CA, United States of America), donated by A. Gritti (San Raffaele Telethon Institute for Gene Therapy, Milan, Italy), were used as breeder pairs to generate homozygous (HOM_Twi) and HET_Twi mice. HOM_Twi, wild type (WT_Twi), HET_Twi, and WT animals were all used for the experiments, and the HET_Twi littermates were also retained for colony maintenance. Control C57BL/6JOlaHsd (Envigo RMS srl) (WT_Ctrl) mice were used to maintain and renew the colony through HET_Twi × WT_Ctrl mating. The animals were maintained under standard housing conditions and used according to the protocols and ethical guidelines approved by the Ministry of Health (permit no. Codice 535/2018-PR; official starting date: 9 July 2018). The mouse body weight (BW) of each animal was measured in grams.

All of the biological samples (tail and brain) were extracted from mice used for other experiments, in line with the 3R principle (replace, reduce, and refine). The animals were deeply anesthetized with a urethane solution (0.8 mL/Hg; Sigma-Aldrich, St. Louis, MO, USA) and euthanized by transcardial perfusion with PBS to remove the blood from the vessels. Subsequently, the tail biopsies were performed and the brains were extracted and placed in a RIPA lysis buffer (R0278–50ML, Sigma-Aldrich) containing a protease and phosphatase inhibitor cocktail (cOmplete (4693116001) and PhosSTOP (4906845001), Roche Diagnostics, Basel, Switzerland). Tail biopsies were performed at PND 14–17 and were used for the DNA isolation, whereas the brains put in the lysis buffer were processed to perform the Galc enzymatic assay. All of the procedures were conducted with maximal efforts to minimize the suffering of the mice.

### 2.2. DNA Extraction from Tail Biopsy

For the pilot study, DNA samples were obtained from 20 Twi and 10 control groups (C57BL/6JOlaHsd mice). For the verification study, DNA samples were obtained from control donors (*n* = 30) and 120 Twi mice.

In the pilot study and in the verification study, DNA isolation was performed using the purification kit (Euroclone spinNAker Universal Genomic DNA mini kit EMR603050) based on spin-minicolumns equipped with membranes that can rapidly and efficiently purify high-quality genomic DNA from solid tissues. The total DNA yield was determined and assessed for purity by using spectrophotometry (DNA with a 260/280 ratio of 1.7 or greater) and checking the DNA quality through the visualization of the intact band by agarose gel electrophoresis (0.7%).

### 2.3. Genotyping of Galactosylceramidase (Galc) in the Twitcher (Twi) Mouse: Novel Protocol

On the extracted and purified DNA, we developed a real-time PCR-based genotyping method using allelic discrimination assays. This method is based on the presence of two primers and two probes in each reaction mix, allowing for the detection of variants in a single nucleic acid sequence [10]. In detail, the victoria (VIC^®^) fluorescent dye detector is a perfect match to the wild type (allele G), and the other FAM^TM^ fluorescent dye detector is a perfect match to the mutation (allele A). Data were collected at the end of the PCR process, allowing us to classify unknown samples: homozygosity for allele G (as WT), homozygosity for allele A (as HOM), and heterozygosity for allele G and allele A (as HET).

We used the real-time PCR system 7300 of Applied Biosystems (Thermo Fisher Scientific, Waltham, MA, United States of America), but other machines were commercially available and compatible with our protocol. Using this machine, we chose to buy all of the products from Applied Biosystems. In detail, we used the TaqPath™ ProAmp™ Master Mix with ROX^TM^ (Catalog number: A3086510) and Custom TaqMan^®^ SNP Genotyping Assay for the *Galc* gene in the Twi mouse model.

The Master Mix is composed of DNA Polymerase, which allows for pre-mixing of PCR reagents at room temperature, as the polymerase is activated after a brief hold step at 95 °C, deoxynucleotide triphosphates (dNTPs), passive reference dye (ROX), i.e., a normalizer of fluorescence fluctuations due to changes in concentration or volume, and optimized buffer components. For further and more detailed information, see the “TaqMan^®^ SNP Genotyping Assays User Guide (Pub. No. MAN0009593)” [11].

The Custom TaqMan^®^ SNP Genotyping Assay (product code: 4332077, assay ID: ANU7GGD, Thermo Fisher Scientific, Massachusetts, United States of America) contains two unlabeled PCR primers: forward and reverse (at 900 nM final concentration), one Applied Biosystems VIC dye, an MGB-labeled probe that detects the Allele G sequence (at 200 nM final concentration), and one Applied Biosystems FAM dye, an MGB labeled probe that detects the Allele A sequence (at 200 nM final concentration). The sequences were designed by Terrell and co-workers [12] and are reported in Table 1.

After receiving the material, we started to perform PCR for the genotyping experiments. First, we prepared the PCR reactions containing three components, i.e., the TaqPath^TM^ ProAmp^TM^ Master Mix, the Custom TaqMan^®^ SNP Genotyping Assay, and nuclease-free water, including 10% overage. Then, we mixed the components and added the appropriate volume of each reaction to each well of an optical plate (catalog number HSS9641, Bio-Rad, Ahracles, CA, United States of America) (48, 96 or 384-well plate, according to available instrument). In each well, we added the same concentration of genomic DNA or nuclease-free water in the no template control (NTC) reaction. The NTC reaction contained all of the reaction components (master mix, assays, water), except the DNA sample, and could be used to identify PCR contamination.

In detail, the procedure to genotype a litter was split into two steps: Step 1, set up the PCR reactions (timing ~15–20 min), and Step 2, set up and run the real-time PCR instrument (timing ~2 h).

#### 2.3.1. Step 1: Set Up the PCR Reactions

1. Calculate the number of reactions to be performed for each assay. Include at least a duplicate of each sample, NTCs, and if possible, known genomic DNA controls (1 WT, 1 HOM, and 1 HET) on each reaction plate. All DNA samples are assessed in a triplicate PCR reaction, plus three NTCs, as well as one animal HOM, one HET, and one WT always in triplicate.
2. Gently swirl the bottle of the TaqPathTM ProAmp^TM^ Master Mix with ROX^TM^ to resuspend it. Caution: TaqPath^TM^ ProAmp^TM^ Master Mix with ROX^TM^ contains glycerin. In case of skin and eye contact, rinse with water for several minutes. Read the material safety data sheet (MSDS) and follow the handling instructions. Wear appropriate protective eyewear, clothing, and gloves.
3. Briefly, vortex and centrifuge the Custom TaqMan^®^ SNP Genotyping Assay for the *Galc* gene (40X). Caution: Custom TaqMan^®^ SNP Genotyping Assay contains formamide. Exposure causes eye, skin, and respiratory tract irritation. It is a possible developmental and birth defect hazard. Read the MSDS and follow the handling instructions. Wear appropriate protective eyewear, clothing, and gloves.
4. Prepare the mix (without DNA) for the number of reactions according to Table 2, plus 10% overage into a microcentrifuge tube. Cap the tube.
5. Mix the components thoroughly, then centrifuge briefly to spin down the contents and eliminate air bubbles.
6. Pipette 5.5 μL of the reaction and mix into each well of an optical plate.
7. Dilute 1 to 20 ng of each purified genomic DNA sample into nuclease-free water for a total sample volume of 4.5 μL (DNA concentration between 0.2–4.4 ng/μL). The final DNA concentration must be at least 0.2 ng/μL for each well reaction. Add 4.5 μL of the DNA of each animal in duplicate and in two wells add 4.5 μL of nuclease-free water for the NTC control. In our prototype experiment, we used 1 ng/μL. If using crude lysates, the sample volume must be up to 25% of the total reaction volume. Once the protocol is established, a fixed volume (as 1 μL) of the DNA sample can be used to simplify the procedure and reduce the time for genotyping as the qualitative part of the procedure. Critical: change tips between each sample to prevent cross-contamination.

#### 2.3.2. Step 2: Set up and Run the Real-Time PCR Instrument

We covered the plate with an optical adhesive cover (catalog number MSB1001, Bio-Rad, California, United States of America) and centrifuged it briefly to spin down the contents and eliminate air bubbles. Pause point: We stored the reaction plate for up to 72 h at room temperature. Then, we loaded the reaction plate in the real-time PCR instrument. We created the optimized protocol (Table 3) following the guidelines of our real-time PCR instrument “Allelic Discrimination Getting Started Guide, Applied Biosystems” [10] and “TaqPath^TM^ ProAmp^TM^ Master Mixes User Guide” [13].

The protocol started with the Pre-read (Step 1) run, in which the instrument recorded the background fluorescence of each well of the plate before the amplification PCR (Step 2). Amplification of the target sequences could be run using any thermal cycler, because the allelic discrimination PCR was an end-point assay. During the post-read run (Step 3), the pre-read fluorescence was subtracted from the post-read fluorescence to account for the pre-amplification background fluorescence, ensuring accurate results. The data were reported as a normalized reporter (R_n_), i.e., the ratio of the fluorescence intensity of the reporter dye signal to the fluorescence intensity of the passive reference dye signal [10].

After the post-read, we directly analyzed the raw data with 7300 SDS software (Applied Biosystem, Thermo Fisher Scientific, Massachusetts, United States of America) that was able to create the allelic discrimination scatter plot of Allele G (axis x) versus Allele A (axis y). From this plot, we derived the genotype of the sample in each well: animals homozygous for Allele G (WT) were localized in the bottom right corner of the graph, animals homozygous for Allele A (HOM) were localized in the top left corner of the graph, animals heterozygous for Alleles A and G (HET) were localized on the diagonal, and NTC samples were localized in the lower-left corner.

Raw data can also be exported and analyzed with other programs such as GraphPad or Excel.

### 2.4. Genotyping Protocol for Galactosylceramidase (Galc) in the Twitcher (Twi) Mouse: The Most Widely Used Protocol

Since 1997, approximately 160 original articles have been published using Twi mice and in almost all of these, the genotyping method that is conventionally used is that proposed by Sakai and co-workers in 1996 [6]. Until now, it was also used by our group [14,15,16,17]. In detail, genomic DNA was extracted from clipped tails, as reported in Section 2.2, and PCR was performed to obtain a genomic DNA fragment around the SNP. PCR fragments were then subjected to restriction enzyme (RE) digestion with EcoRV and subsequently to agarose gel electrophoresis. RE digestion analysis was used for Twi mice genotyping.

### 2.5. Galc Enzymatic Activity Assay

The Galc enzymatic activity assay was carried out in the brain. Tissues were lysed on ice with a radioimmunoprecipitation assay (RIPA) buffer (Sigma-Aldrich, Missouri, United States of America) containing a protease and phosphatase inhibitor cocktail (cOmplete and PhosSTOP, Roche Diagnostics, Basel, Switzerland). Tissue lysates were homogenized in 1.5 mL of Eppendorf tube with appropriate micropestles. Then, after centrifugation (15,000× *g* for 25 min at 4 °C), the supernatants were collected and tested for protein concentration using the micro-bicinchoninic acid (BCA) Protein Assay Kit (Thermo Fisher Scientific, Massachusetts, United States of America). To measure the Galc activity, we used 6-hexadecanoylamino-4-methylumbelliferyl-b-D-galactopyranoside (HMU-bGal, Biosynth Carbosynth, Compton, UK), the fluorescent substrate currently used for the clinical diagnosis of KD [18]: 10 μL of lysates was added to 20 μL of 50 mM HMU-bGal substrate solution, which was incubated for 17 h at 37 °C. The reaction was then stopped, and the fluorescent product [4-methylumbelliferone (4-MU)] was read by using the microplate GloMax fluorescence reader. The Galc activity (reported as nanomole per milligram protein extract after 17 h of incubation) was calculated by comparison with a standard curve previously obtained by measuring the fluorescence of different concentrations of 4-MU.

### 2.6. Motor Behavioral Experiment: Rotarod Test

The Rotarod test was carried out every 5 days from PND 25–28. As was previously done, a rotarod apparatus (Ugo Basile, Varese, Italy) was used to assess the motor coordination skills [14].

The rotarod apparatus consisted of five 3 cm diameter cylinders, which were suitably machined to provide grip. Six 25 cm diameter dividers made five lanes, each 5.7 cm wide, enabling five mice to be on the rotor simultaneously. The mice were placed on the rotating rod suspended horizontally at a height of 16 cm from the floor. Four trials per day with an inter-trial interval (ITI) of 15 min and a cut-off of 5 min at 4 rpm speed were performed for each animal and the mean latency to fall was calculated.

### 2.7. Statistical Analysis

Data were statistically analyzed using Prism 6.00 (GraphPad Software, San Diego, CA, United States of America; RRID:SCR_002798). For the parametric data, Student’s t-test (unpaired, two-tailed), one-way analysis of variance (ANOVA) (Tukey’s multiple comparisons test), or linear regression was used; the mean values obtained in each repeated experiment were assumed to be normally distributed about the true mean. Statistical significance refers to results for which *p* < 0.05 was obtained.

## 3. Results

### 3.1. Genotyping Protocol for Galactosylceramidase (Galc) in the Twitcher (Twi) Mouse: A Pilot Study

**Figure 1:**
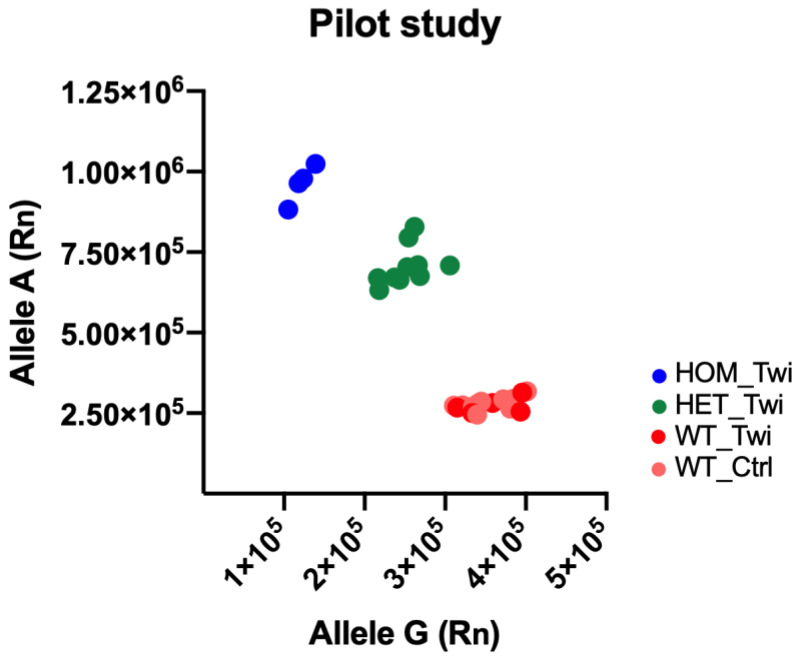
Genotyping of *galactosylceramidase* (*Galc*) SNP c.355G>A in the Twitcher (Twi) mouse. The Scatter plot of Allele G (axis x) versus Allele A (axis y) allowed us to identify the presence of the SNP in each animal. Animals homozygous for Allele G (WT) were localized in the bottom right corner of the graph (red and pink circles), animals homozygous for Allele A (HOM) were localized in the top left corner of the graph (blue circles), and animals heterozygous for Alleles A and G (HET) were localized on the diagonal (green circles).

Initially, different mice’s DNA samples were used to assess whether allele-discrimination real-time PCR could detect SNP c.355G>A. We performed a pilot study to assess the SNP in Twi mice compared with the control animals (Twi mice: *n* = 20; control mice: *n* = 10). We carried out the following steps: (i) Set up the PCR reactions as reported in Point 2.3.1 of the Materials and Methods, (ii) load the plate in the real-time PCR detection system, (iii) set up and run the pre-read as reported in Step 1 of Table 3, (iv) set up and run the amplification as reported in Step 2 of Table 3. Step 2 could be run by any other thermal cycler, (v) set up and run Post-read as reported in Step 3 of Table 3, (vi) evaluate the results, (vii) create the allelic discrimination scatter plot of Allele G (axis x) versus Allele A (axis y). The results were directly plotted using the SDS software on a scatter plot of Allele G (axis x) versus Allele A (axis y). The clustering of points varied along the following: the horizontal axis Allele G, WT animals; the vertical axis Allele A, HOM animals; or diagonal Allele G/Allele A, HET animals. The raw data of the fluorescence variation after PCR amplification were plotted, as shown in Figure 1. The plot of the results of the genotyping experiment showed ten points as being HET_Twi mice (green), 4 points as being HOM_Twi (blue), 16 points as being WT (10 from the group of control WT_Ctrl (pink), and 6 as being Twi mice, WT_Twi (red).

**Figure 2:**
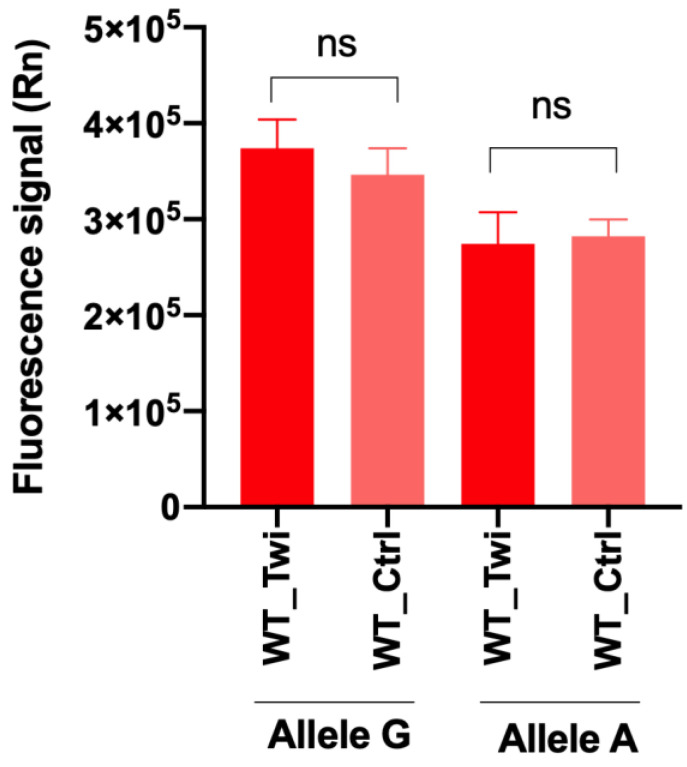
The fluorescence signal of Allele G and Allele A in the two groups of WT mice, WT_Twi compared to WT_Ctrl. Student’s t-test analysis, ns = not significant.

As reported in Figure 2, we did not observe a significant variation in the mean of the fluorescence signal of both alleles, G and A, in WT_Twi compared to WT_Ctrl, indicating the high fidelity of the allele-discrimination real-time PCR for the detection of SNP at position 355 of the *Galc* gene.

### 3.2. Galc Enzymatic Activity (HMU Assay) Corroborated the Mice Identification with the New Protocol of Genotyping

**Figure 3:**
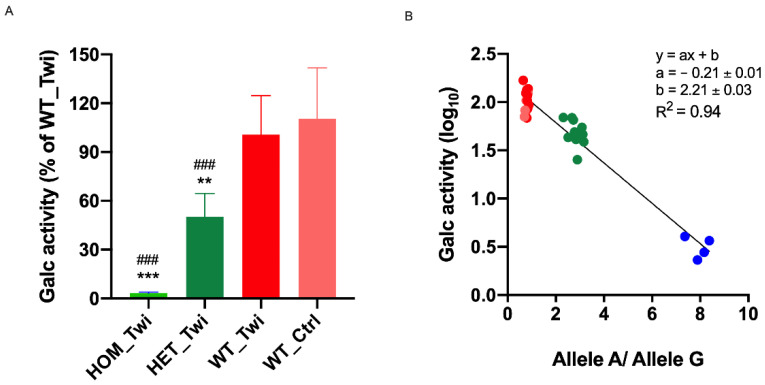
(**A**) Brain Galc activity. Galc activity was assayed in the extracted brain using the HMU-bGal assay. Means ± SEM (HOM_Twi *n* = 4; HET_Twi *n* = 10; WT_Twi *n* = 6 and WT_Ctrl *n* = 10) ** *p* < 0.01 HET_Twi versus WT_Twi, *** *p* < 0.001 HOM_Twi versus WT_Twi, ### *p* < 0.001 HOM_Twi versus WT_Ctrl and ### *p* < 0.001 HET_Twi versus WT_Ctrl, one-way ANOVA (Tukey’s test). (**B**) The Galc activity (log10) versus the ratio Allele A/Allele G (Rn / Rn). Data are superimposed to a linear fit (parameters of the linear regression are given in the figure inset). Animals homozygous for Allele G (WT) were localized in the top left corner of the graph (red and pink circles), animals homozygous for Allele A (HOM) were localized in the bottom right corner of the graph (blue circles), animals heterozygous for Alleles A and G (HET) were localized on the diagonal (green circles).

To validate our innovative protocol for *Galc* genotyping, we evaluated the GALC enzymatic activity in the brain of Twi mice of the pilot study (Twi: *n* = 20) and 10 animals from the control group. In the animals identified as HOM_Twi, we highlighted the lack of enzymatic activity. We detected a significantly higher activity in the brain in both WT_Ctrl and WT_Twi (Figure 3A), without significant differences between the two groups.

Remarkably, a strong correlation was observed between the GALC activity and the ratio Allele A/Allele G (Rn/Rn, Figure 3B). In addition, in this case, the graph shows a clear clustering of the different mouse genotypes.

### 3.3. Genotyping Galactosylceramidase (Galc) in the Twitcher (Twi) Mouse: Verification Study

**Figure 4:**
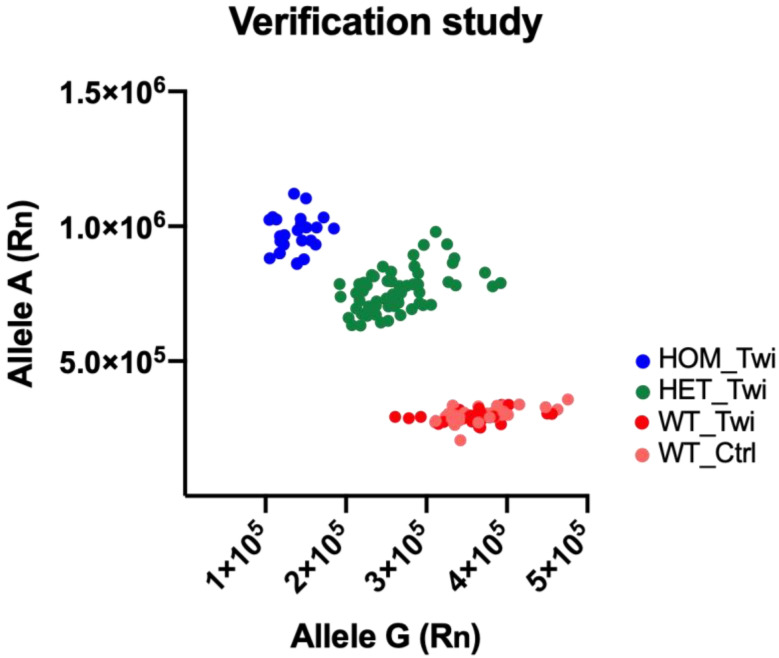
Genotyping of galactosylceramidase (*Galc*) SNP c.355G>A in 120 Twitcher (Twi) mice and 30 control mice (Ctrl) for the verification study, cohort A. The scatter plot of Allele G (axis x) versus Allele A (axis y) allowed us to identify the presence of the SNP in each animal. Animals homozygous for Allele G (WT) were localized in the bottom right corner of the graph (red and pink circles), animals homozygous for Allele A (HOM) were localized in the top left corner of the graph (blue circles), and animals heterozygous for Alleles A and G (HET) were localized on the diagonal (green circles).

Utilizing a Twi cohort comprising of 120 animals, SNP c.355G>A detection in DNA was assessed by allele-discrimination real-time PCR and compared with 30 control mice. As shown in Figure 4, all 30 mice of the control group were WT and c.355G>A was identified in 29 animals of the TWI group (HOM). Thus, we detected 29 WT_Twi, 62 HET_Twi, and 29 HOM_Twi in the colony of the Twitcher mouse. These data demonstrate that the protocol for *Galc* genotyping detected the presence of SNP c.355G>A in a fast, unambiguous way, taking less than three hours including DNA isolation and purification.

**Figure 5:**
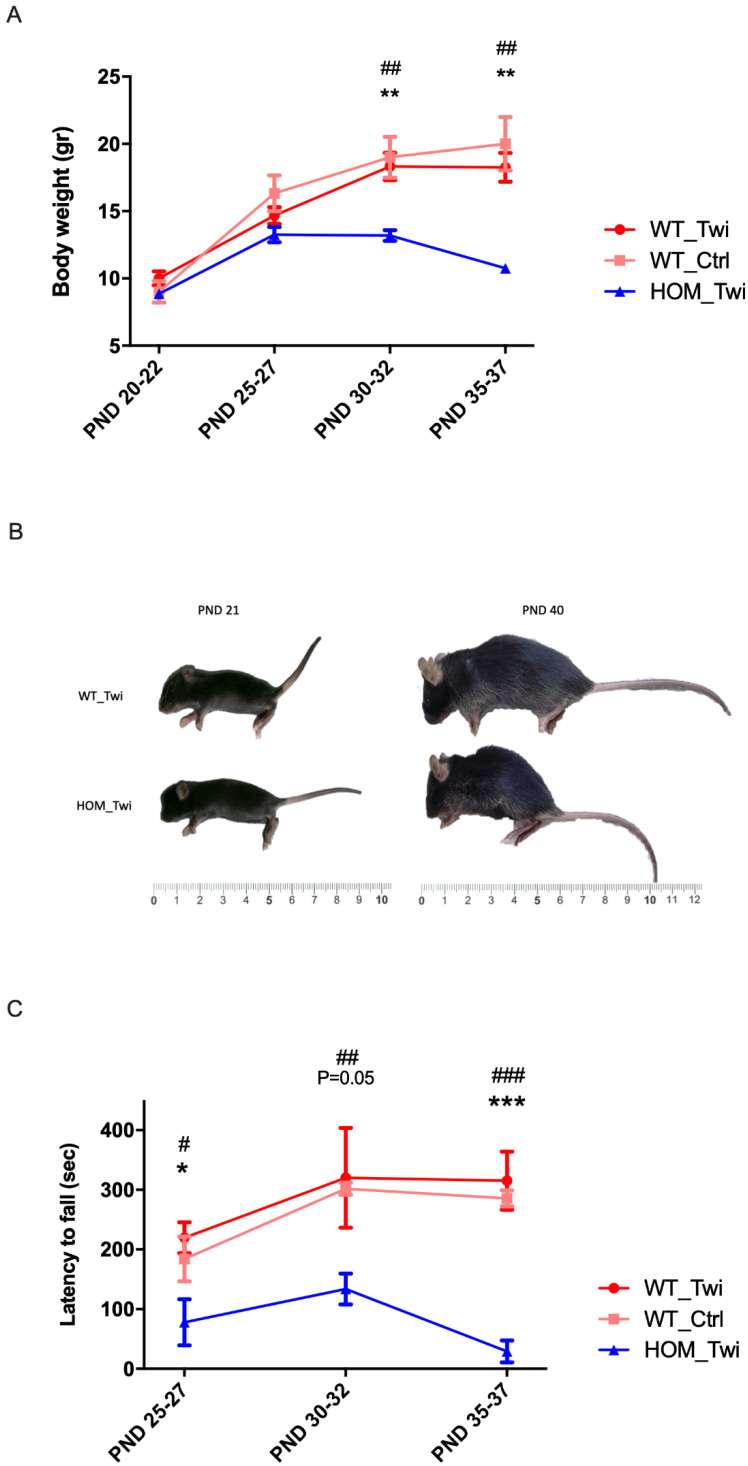
(**A**) Body weight versus post-natal day (PND). Body weight (BW) was measured (in grams) every 5 days. ** *p* < 0.01 WT_Twi versus HOM_Twi; ## *p* < 0.01 WT_Ctrl versus HOM_Twi. (**B**) Representative image of WT_Twi (top) versus HOM_Twi mouse (bottom) at PND 22 and PND 40. (**C**) The Rotarod test was performed on HOM_Twi, WT_Twi, and WT_Ctrl mice every 5 days. Data are reported in the graph in seconds (s). * *p* < 0.05, *** *p* < 0.001 WT_Twi versus HOM_Twi; # *p* < 0.05, ## *p* < 0.01, ### *p* < 0.001 WT_Ctrl versus HOM_Twi. Data are reported as mean ± SEM and compared using a T-test.

To confirm the development of the Twi phenotype in mice identified as HOM_Twi, we monitored the Twi growth between PND 22 and PND 37. As reported in Figure 5A and 5B, the HOM_Twi mice appeared essentially normal until approximately PND 28, when their growth slowed down and death occurred by ~40 days of age. As expected, the body weight (BW) of HOM_Twi mice did not show the physiological BW increase found for WT_Twi and in WT_Ctrl mice. Figure 5B visually showed the forfeit of the weight of HOM_Twi and the characteristic hunched back compared to WT_Twi mice.

To evaluate the motor function, we performed the rotarod test, commonly used to evaluate motor coordination in rodents (Figure 5C). As expected, WT_Twi mice presented a rotarod performance comparable with the group of WT_Ctrl, whereas HOM_Twi performed significantly worse than all of the WT mice.

Altogether these data confirm that HOM_Twi mice go on to develop the Twitcher phenotype, further corroborating the correct genotyping with the new protocol.

### 3.4. Comparison with the Mainly Used Protocol for Twitcher Genotyping

**Figure 6:**
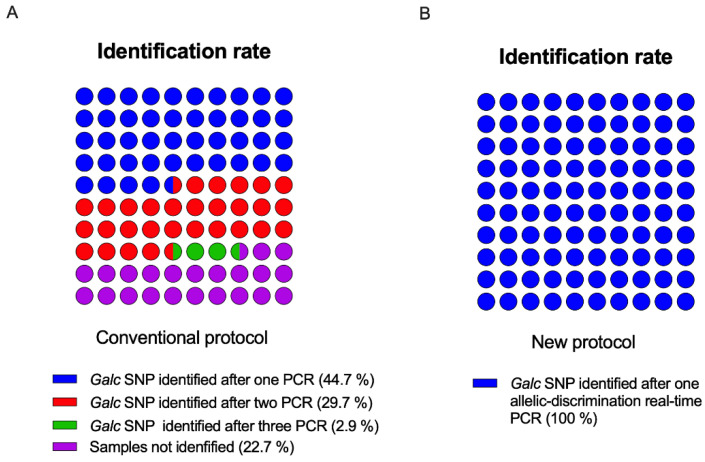
Identification rate of *Galc* SNP in the Twitcher (Twi) mouse using the conventional protocol (**A**) and of the new protocol based on the allele-discrimination real-time PCR (**B**).

To quantitatively compare our protocol with the most widely used one (Sakai’s protocol [6]), genomic DNA isolated from 143 clipped tails of Twi mice was processed using both methods. Following Sakai and co-workers’ procedure, 44.7% of the DNA samples were identified after the first PCR, 29.7% after the second PCR, and 2.9% after the third PCR. The *Galc* mutation status was not identified in 22.7% of the animals. Using the allele-discrimination PCR, all of the 143 samples were identified using the first analysis (100%) and no animals were not genotyped (Figure 6). Finally, it is worth noting that the proposed method, which is much faster than the traditional one, allowed for better planning of the experiments and easier colony management.

Moreover, the procedure used in the conventional protocol is a time-consuming (≅30 h for a total genotyping experiment) and labor-intensive process that sometimes yields ambiguous results. For these reasons, conventionally, enzymatic activity measurement in KD tissues is commonly associated with RE digestion of the amplified *Galc* gene segment, substantially increasing the laboratory activity needed to characterize each mouse.

Concerning the specific costs, the DNA isolation costs are the same for the two methods (~3.5 EUR/mouse), while the cost of each genotyping experiment with allele-discrimination PCR is ~4 EUR compared with the 3 EUR for Sakai’s protocol. However, considering that only around half of the mice can be successfully genotyped after the first experiment with Sakai’s protocol, the need to repeat the genotyping one or two additional times increases the effective cost for every single mouse up to 9 EUR.

Compared with these abovementioned multi-step protocols, our method based on allele-discrimination real-time PCR for Twi genotype determination used a single-step protocol, considerably increasing the accuracy and speed in the genotyping discrimination.

## 4. Discussion

In this article, we set-up and demonstrated a novel protocol for genotyping the *Galc* gene in the Twi mouse through allele discrimination RT-PCR. We demonstrated that this method distinguished accurately and without ambiguity between HOM, WT, and HET animals from genomic DNA. The protocol is faster and less work-consuming in respect to the standard PCR protocol (≈3 h versus ≈ 30 h) and all animals are correctly genotyped in the first analysis without ambiguous results and/or no animals not being genotyped.

We initially conducted a pilot study in 20 Twi mice and 10 control mice, identifying 4 HOM_Twi, 10 HET_Twi, and 16 WT (WT_Twi *plus* WT_Ctrl). Parallelly, in the same cohort, we also evaluated the activity levels of the Galc enzyme with the HMU-bGal assay, the fluorescent substrate currently used for the clinical diagnosis of Krabbe disease [18], and highlighted the expected lack of Galc enzymatic activity [2,6] in all of the identified HOM_Twi, thus confirming the results of the novel genotyping protocol. As expected, no difference in the fluorescence signal of the *Galc* alleles (i.e., Allele A and Allele G) was detected within the WT groups, WT_Twi and WT_Ctrl.

Given the very promising data, we proceeded with testing the ability of allele-discrimination PCR genotyping protocol in an independent verification study. We conducted a verification study in 120 Twi mice and 30 control mice, identifying 29 HOM_Twi, 62 HET_Twi, and 59 WT (WT_Twi *plus* WT_Ctrl). The verification study revealed that the protocol was able to correctly identify HOM_Twi, HET_Twi, WT_Twi, and WT_Ctrl in an accurate, sensitive, easy, and unambiguous way. To confirm the development of the Twi phenotype in the mice identified as HOM_Twi, we monitored the HOM_Twi compared to WT_Twi and WT_Ctrl. We observed that the HOM_Twi mice appeared essentially normal at birth but at ~28 days of age their growth slowed down and death occurred by ~40 days of age. Regarding muscular function, the rotarod test highlighted a strong reduction in the motor performance in HOM_Twi, a feature widely reported in the literature [14], and no variations between WT_Twi and WT_Ctrl. Thus, we confirm that HOM_Twi mice go on to develop the Twi phenotype, with a life span and a decrease in muscular function in line with that known for KD [2,19,20].

Overall, this new protocol based on allele-discrimination PCR represents an attractive option for an easy, fast and robust individuation of the nucleotide substitution (G>A) in the *Galc* gene in the Twi mouse model.

Ever since we developed this new genotyping protocol, HET_Twi animals have been used to maintain our colony. In the past year, we had about 50 births from HET_Twi x HET_Twi mating, with the birth of HOM_Twi animals in each mating, confirming that the correct genotyping carried out.

Given the very interesting data, we proceeded with comparing our genotyping protocol with the most widely used protocol. Since 1997, approximately 160 original articles have been published using Twi mice and in almost all of these, the genotyping method used was that proposed by Sakai and co-workers in 1996 [6]. In detail, genomic DNA was extracted from clipped tails and PCR was performed to obtain a genomic DNA fragment around the SNP. PCR fragments were then subjected to RE digestion with EcoRV and subsequently to agarose gel electrophoresis. RE digestion analysis has traditionally been used for Twi mice genotyping. However, the procedure is a time-consuming (≈ 30 h for a total genotyping experiment) and labor-intensive process that sometimes yields ambiguous results. Indeed, inconclusive genotyping is a factor that can impact preclinical studies and basic research reproducibility, contributing to the “reproducibility crisis” [6,21]. For these reasons, conventionally, the enzymatic activity measurement in KD tissues is commonly associated with RE digestion of the amplified *Galc* gene segment, substantially increasing the laboratory activity needed to characterize each mouse. Our data showed that by using the allele-discrimination PCR, all of the samples were identified by the first analysis, without ambiguous results. Finally, it is worth noting that the proposed method, being much faster than the traditional one, allows for better planning of the experiments and easier colony management.

In 2007, Terrell and collaborators published an innovative approach to genotype several mammalian models of KD [12]. The procedure presented is a PCR followed by fluorescence analysis in a plate reader. The protocol has not been widely used by researchers working with Twi, probably due to the advent of real-time PCR instruments. Indeed, following the development of real-time PCR, the use of two separate instruments, a thermocycler and fluorescence plate reader, has decreased. Nevertheless, the molecular beacons designed by Terrell and co-worker have a strong affinity for the template of Galc, indeed we used the same sequences of molecular beacons in our study.

Recently, another alternative method based on high-resolution melting (HRM) analysis was published for Galc genotyping in Twi mice by Signorini and collaborators [22]. Differences in melting curves and in the melting temperature (Tm) between HOM and HET genotypes can predict the phenotypes in Twi based on single-nucleotide polymorphisms. The HRM analysis is considered to be a rapid, closed-tube, and cost-efficient approach for genotyping. Nevertheless, the necessity of reference curve-based targeted genotyping can affect the performance and data analysis of HRM results, as well as the different isolation methods and the sample compositions making parallel analysis difficult [23]. Thus, HRM analysis [22] and molecular beacon assay [12] represent other options for researchers working in the field of KD.

In conclusion, with this study, we developed and demonstrated a novel genotyping protocol based on allele discrimination RT-PCR for distinguishing accurately, timely, and without ambiguity between HOM_Twi, WT_Twi, and HET_Twi mice. The Twi mouse serves as the primary preclinical animal model for identifying disease mechanisms and evaluating therapeutic interventions for KD, thus the genetic status of Twi mice is routinely analyzed in every research group worldwide following a research project on KD. In this context, this novel genotyping protocol is expected to improve the routine of every researcher working with Twi and studies in KD.

## Figures and Tables

**Table 1:** Information necessary to order a Custom TaqMan^®^ SNP Genotyping Assay for the *Galc* gene in the Twi mouse model.

Name	Sequences
Forward Primer	CTGCTTAGAATCAATCAGACTG
Reverse Primer	CTCAACAACGGACAATTACC
Probe 1 (allele G)-VIC-MGB Dye	CCAGGCTGGTATTACCTGG
Probe 2 (allele A)-FAM-MGB Dye	CCAGGCTGATATTACCTGG
Context Sequence	AAATGAGTTGGTCCGAC[C/T]ATAATGGACTTCTGTCAC

**Table 2:** Components and volume of the PCR reactions.

Component	Volume per Reaction	Volume per Reaction Plus 10% Overage
TaqPathTM ProAmp^TM^ Master Mix with ROX^TM^	5 μL	5.5 μL
Custom TaqMan^®^ SNP Genotyping Assay for *Galc* gene (40X)	0.25μL	0.275 μL
Nuclease-free water	0.25 μL	μL

**Table 3:** Optimized real-time PCR protocol for *Galc* allelic discrimination.

Step Number	Step	Temperature	Time	Cycles
1#	Pre-read	60 °C	30 s	Hold
2#	Initial denature / Enzyme activation	95 °C	5 min	Hold
	Denature	95 °C	15 s	40
	Anneal/Extend	60 °C	1 min	
3#	Post-read	60 °C	30 s	Hold

# Remember to adjust the sample volume.

## Data Availability

Datasets generated during the study are freely available on the DRYAD platform (www.datadryad.org, accessed on 1 December 2022), at the following link: https://doi.org/10.5061/dryad.k3j9kd5br [24].
